# Dominance of *Dermacentor reticulatus* over *Ixodes ricinus* (Ixodidae) on livestock, companion animals and wild ruminants in eastern and central Poland

**DOI:** 10.1007/s10493-015-9889-0

**Published:** 2015-02-26

**Authors:** Ewa J. Mierzejewska, Renata Welc-Faleciak, Grzegorz Karbowiak, Maciej Kowalec, Jerzy M. Behnke, Anna Bajer

**Affiliations:** 1 Department of Parasitology, Institute of Zoology, Faculty of Biology, University of Warsaw, 1 Miecznikowa Street, 02-096 Warsaw, Poland; 2 W. Stefański Institute of Parasitology of the Polish Academy of Sciences, Twarda 51/55, Warsaw, Poland; 3 School of Life Sciences, University of Nottingham, University Park, Nottingham, NG7 2RD UK

**Keywords:** *Dermacentor reticulatus*, *Ixodes ricinus*, Hosts, Abundance, Engorgement, Dogs, Livestock, European bison

## Abstract

The most common tick species parasitizing animals in Poland are *Ixodes ricinus* and *Dermacentor reticulatus*. These tick species differ in their distribution, habitats, seasonal activity and host specificity. *Ixodes ricinus* is the most prevalent and widely distributed, whereas the range of *D. reticulatus* is limited to eastern and central parts of the country with several new foci in the middle-west and the west. However, as in many central European countries, the range of *D. reticulatus* is expanding, and some authors have correlated this expansion with an increasing number of available hosts. The aim of the present study was to determine the tick fauna on domestic and livestock animals in two areas endemic for *I. ricinus* and *D. reticulatus* and to compare the risk of infestation with different tick species in open and forest areas. Over a 14 month period, 732 ticks were collected from five host species including domestic animals (dogs and cats), livestock (cows and horses) and wildlife (European bison) in two areas, central and NE Poland, endemic for *D. reticulatus*. Three tick species were recorded: *D. reticulatus* (623 individuals; 85.1 % of all collected ticks), *I. ricinus* (106 individuals; 14.5 %) and three females of *Ixodes hexagonus* (0.4 %) from a dog. *Dermacentor reticulatus* was the dominant tick species found on four host species and constituted 86, 81, 97 and 100 % of all ticks from dogs, horses, cows and bison, respectively, and was collected from animals throughout the year, including during the winter. The common tick, *I. ricinus,* was the dominant tick collected from cats (94 %). Fully-engorged, ready-for-reproduction females of *D. reticulatus* were collected from all host species. In May 2012, questing ticks were collected by dragging in forest or open habitats. The density of adult marsh ticks in open areas was around 2 ticks/100 m^2^ in the majority of locations, with a maximum of 9.5 ticks/100 m^2^. The density of adult *I. ricinus* was much lower in its typical habitat (forests: range 0.8–2.2 ticks/100 m^2^) between three and seven times lower than the density of *D. reticulatus* in its typical habitat. In regions endemic for marsh ticks, this tick species constitutes the main risk of tick infestation for livestock and dogs throughout the year. Livestock and companion animals are competent hosts for *D. reticulatus*, enabling the completion of the tick’s life cycle. Anti-tick treatment should be adjusted to marsh tick seasonal activity and drug sensitivity.

## Introduction

In Poland 19 species of ticks are known to occur as the established tick fauna of the region. Nine species parasitize domestic and farm animals (Nowak-Chmura and Siuda 2012) and among these the most common species of hard ticks are *I. ricinus* and *D. reticulatus*. Both tick species are vectors of *Borrelia burgdorferi* s.l. and the tick-borne encephalitis virus (TBEV), pathogens that have major significance in human and veterinary medicine (Zygner et al. 2008; Bonnet et al. 2013; Mierzejewska et al. 2013; Reye et al. 2013). They can also transmit *Rickettsia* spp. and *Anaplasma phagocytophilum* (Zygner et al. 2008; Bonnet et al. 2013; Wójcik-Fatla et al. 2013)*. Dermacentor reticulatus* is the main vector of *Babesia canis,* the etiological agent of canine babesiosis. This disease constitutes the most important infectious disease of dogs in regions of Poland endemic for *D. reticulatus* (Bajer et al. 2014b).

*Ixodes ricinus* and *D. reticulatus* differ in distribution, habitats, seasonal activity and host specificity (Nowak-Chmura and Siuda 2012). *Ixodes ricinus* is the most prevalent and widely distributed tick species in Poland, while the range of *D. reticulatus* is limited to eastern and central parts of the country with several new foci in the middle-west and the west (Nowak 2011; Mierzejewska et al. 2012, 2014). The typical habitats of *I. ricinus* include deciduous, mixed and coniferous woodland, heathland, moorland, rough pasture and urban parks. This tick is most active from May to early October and has a very wide range of hosts: lizards, many species of birds, small, medium-sized and large mammals and humans (Medlock et al. 2013; EFSA 2010). In contrast, *D. reticulatus* inhabits open areas such as fallow lands, river banks and lake shores covered with tall grasses and shrubs, edges of wetlands, shrubby pastures and forest paths (Bogdaszewska 2004; Bajer et al. 2014a; Zygner et al. 2009). It first appears early in spring and following summer diapause is again active in late autumn/early winter until the first snowfall. The main hosts are believed to be large mammals, mainly elks, red deer, cattle and dogs (Karbowiak 2009).

Although tick infestations on domestic and farm animals have been well studied, data about tick engorgement levels on different hosts under natural conditions or about competency of hosts for particular tick species are still scarce. Engorgement level plays a crucial role for the next off-host phase of the life cycle (molting of instars or oviposition in females). In the case of an uncompleted blood meal, there is no possibility for the completion of the tick’s life cycle. Thus identifying animal species that support the completion of life cycles of particular tick species helps us to understand the reasons for expansion of this tick species and the environmental circulation of the pathogen linked to the species concerned. In the face of the rapid expansion of the range of *D. reticulatus* and the extent of canine babesiosis in many European countries in recent years, several authors have suggested an association between the expansion and an increasing numbers of suitable hosts, especially red deer, elk or wild boar (Sréter et al. 2005; Dautel et al. 2006; Nijhof et al. 2007; Karbowiak 2009, 2014; Cochez et al. 2012; Beugnet and Chalvet-Monfray 2013). However, the competence of livestock and domestic animals as hosts for *I. ricinus* and *D. reticulatus* has not been verified. In comparison to the overall populations of large mammals in Poland (app. 710,000 roe deer, 150,000 red deer, 180,000 wild boars, 1000 European bisons), the populations of farm and domestic animals are much larger. There are about 5 mln of cattle, 300,000 of sheep, 300,000 horses in Poland. High populations of domestic dogs (about 7–8 mln) and cats (5–6 mln) exist in Poland because almost 60 % of families own a dog or a cat (data from GUS–Main Statistical Office and from pet food industry estimates).

In order to verify the competence of natural hosts for these two tick species, we developed a simple and practical method based on the determination and comparison of body mass of questing and foraging ticks. Then classification of foraging ticks to biologically relevant engorgement classes was conducted, reflecting further opportunity for tick life cycle completion. We predicted that although ticks collected from naturally infested animals can be at different stages of a blood meal (interrupted by tick collection by owner or veterinarian), they should represent a complete range of engorgement stages, including also a significant number of almost full ready-for-reproduction females. We also predicted that in areas endemic for two tick species, *D. reticulatus* should constitute a significant proportion of foraging ticks, due to ‘sharing’ of open habitats (pastures, meadows, urban and peri-urban areas) with potential hosts. Thus the aims of the present study were: (1) to determine the composition of the tick community on livestock and companion animals in two areas endemic both for *I. ricinus* and *D. reticulatus*; (2) to compare the risk of infestation with different tick species in open and forest areas; (3) to monitor seasonal changes in tick communities on hosts and finally (4) to assess the competency of different host species for the completion of the life cycle of *D. reticulatus.*

## Materials and methods

### Abundance of questing ticks in environment

**Fig. 1:**
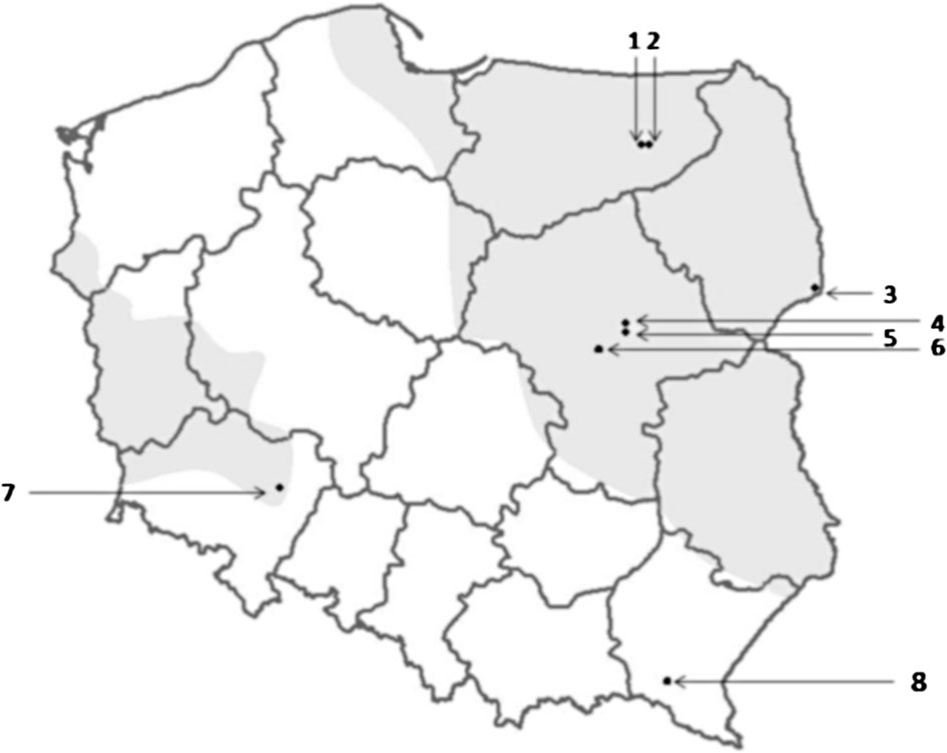
The location of study sites where ticks were collected (filled *diamond shapes* distinguished by *numbered arrows*), superimposed on a map of Poland showing the endemic regions for *Dermacentor reticulatus* (*shaded areas*), as reported by Mierzejewska et al. (2012, 2014) and Nowak (2011). *1.* Urwitałt; *2.* Dziubiele; *3.* Białowieża Primeval Forest; *4.* Tłuszcz; *5.* Dąbrowica; *6.* Warsaw; *7.* Wrocław; *8.* Rymanów

To compare the risk of infestation by *I. ricinus* and adult *D. reticulatus*, the abundance of ticks in the environment was assessed for each location (Mazury Lake District and Mazowsze in central Poland) either in forest or open habitats (Table 2). Questing ticks were generally collected in the same areas as those in which foraging ticks were collected from hosts (Fig. 1). Questing ticks were collected by dragging a wollen blanket (1.2 × 0.8 m) in the forests or in fallow lands in the Mazowsze and Warmińsko–Mazurskie regions in May 2012 (Table 2; Fig. 1). Additionally, ticks were picked from clothing after dragging or directly from vegetation in front of the dragging person, when noted on a dragged transect. Ticks were collected twice a day at the peak of activity between 9–11 a.m. and 4–6 p.m. and were preserved in 96 % ethanol for further identification in the laboratory. After determination of species and sex by stereo microscopy, abundance was calculated and expressed as the number of ticks per 100 m^2^ (Table 3).

### Determination of the mean weight of questing adult *Dermacentor reticulatus* and *Ixodes ricinus*

To determine ‘engorgement success’ of ticks on different hosts, we needed to separate engorged and not engorged ticks collected from a host, and therefore we conducted a preliminary study to determine the body mass of questing adult ticks of both species. Questing ticks were collected both in Mazowsze and Warmińsko-Mazurskie regions to control for any regional variation. In the Mazowsze region 50 ticks of *I. ricinus* (23 females and 27 males) and 50 ticks of *D. reticulatus* (20 females and 30 males) were collected. Ticks were collected in the capital city of Warsaw (fallow land in Siekierki), in two city forests (Kabacki and Bielański forests) (Welc-Faleciak et al. 2014) and in open habitats (meadows, fallow lands) in Stoski, Kury and Dąbrowica villages (30–50 km outside Warsaw). From the Warmińsko-Mazurskie region, traditionally believed to be a region of high risk of tick infestation and tick-borne diseases, 19 *I. ricinus* (9 females and 10 males) and 46 *D. reticulatus* ticks (21 females and 25 males) were used for measurements. Tick abundances were determined on fallow lands in Urwitałt, Stawek and Dziubiele villages situated in the vicinity of the town of Mikołajki (‘summer capital’ of Poland) in Mazury Lake District (Fig. 1). Before weighing, ticks were dried separately and then the body weight of each specimen was recorded with an analytical balance (Radwag, Poland) with accuracy to 1 µg.

### Distribution of tick species on different hosts

In a period of fourteen months (May 2012–June 2013) 586 ticks were collected from domestic (cat, dog) and farm (cow, horse) animals living in regions endemic for the marsh tick *D. reticulatus*, in the Mazowsze and Warmińsko-Mazurskie regions (Table 1a, Fig. 1). Ticks were collected from dogs and cats presenting at veterinary practices for routine health inspection visits in the Mazowsze region (Warsaw and Tłuszcz, a veterinary practice closest to the villages of Kury and Stoski). Additionally, ticks (n = 29) were removed from 11 sled dogs participating for 14 days in September 2012 in a training camp in Urwitałt, in the Mazury Lake District (Table 1a, b). These dogs were treated with acaricide spot-on containing fipronil (Fiprex, Frontline).

**Table 1:** Distribution of tick species on different hosts (a) by region and month of study, (b) mean infestation for selected host groups

Host	Region	Time				Number of ticks									
		Season		Month		D.r. F	D.r. M	D.r. T	I.r. F	I.r. M	I.r. N	I.r. T	I.h. F	I.h. T	∑
(*a*)															
Dog	Mazowsze region	Spring		March		12	7	19	0	0	0	0	0	0	19
				April		13	12	25	3	1	0	4	0	0	29
				May		38	13	51	6	1	1	8	0	0	59
		Summer		June		2	0	2	4	0	0	4	3	3	9
				July, August		5	1	6	9	0	0	9	0	0	15
		Autumn		September		10	5	15	1	0	0	1	0	0	16
				November		3	2	5	0	0	0	0	0	0	5
		Winter		December		2	4	6	0	0	0	0	0	0	6
				January		0	46	46	0	0	0	0	0	0	46
				February		0	1	1	0	0	0	0	0	0	1
	Warmińsko-Mazurskie region	Autumn		September		20	14	34	5	0	0	5	0	0	39
**∑**						105	105	210	28	2	1	31	3	3	244
Cat	Mazowsze region	Spring		April		1	0	1	0	0	0	0	0	0	1
				May		1	0	1	20	3	0	23	0	0	24
		Summer		June		0	0	0	1	0	0	1	0	0	1
				July, August		0	0	0	5	0	1	6	0	0	6
		Autumn		November		0	0	0	1	0	0	1	0	0	1
**∑**						2	0	2	27	3	1	31	0	0	33
Horse	Warmińsko-Mazurskie region	Spring		May		102	54	156	17	5	0	22	0	0	178
		Summer		June		4	0	4	11	1	1	13	0	0	17
				July, August		0	0	0	5	0	0	5	0	0	5
		Autumn		October		11	3	14	0	1	0	1	0	0	15
**∑**						117	57	174	33	7	1	41	0	0	215
Cow	Mazowsze region	Autumn		October, November		53	38	91	3	0	0	3	0	0	94
European bison	Podlaskie region	Winter		December		12	134	146	0	0	0	0	0	0	146

Region	Localization	Host number of individuals	Time (month, season)	No. and mean/ind.						
				D.r. F	D.r. M	D.r. T	I.r. F	I.r. M	I.r. N	I.r. T
(*b*)										
Warmińsko-Mazurskie region	Urwitałt N 53°48′17.29″ E 21°38′25.43″	Horse (n = 6)	May 2012, Spring	102	59	161	12	5	0	17
				17	9.83	26.83	2	0.83	0	2.83
	Dziubiele N 53°48′07.42″ E 21°43′57.08″	Horse (n = 32)	June 2012, Summer	4	0	4	11	1	1	13
				0.13	0	0.13	0.34	0.03	0.03	0.4
		Horse (n = 8)	October 2012, Autumn	11	3	14	0	1	0	1
				1.38	0.38	1.75	0.00	0.13	0.00	0.13
	Urwitałt N 53°48′17.29″ E 21°38′25.43″	Dog (n = 11)	September 2012, Autumn	29	0	29	0	0	0	0
				2.64	0	2.64	0	0	0	0
Mazowsze region	Dąbrowica N 52°42′04.83″ E 23°51′10.80″	Cow (n = 3)	Oct, Nov 2012, Autumn	53	38	91	3	0	0	3
				17.67	12.67	30.33	1	0	0	1
	Tłuszcz N 52°26′04.00″ E 21°25′55.33″	Cat (n = 1)	June, July, Aug 2012, Summer	0	0	0	5	0	1	6
			April 2013, Spring	1	0	1	0	0	0	0
			May 2013, Spring	1	0	1	20	3	0	23
			June 2013, Summer	0	0	0	1	0	0	1
Podlaskie region	Białowieża N 52°42′04.83″ E 23°51′10.80″	European bison (n = 6)	December 2003, Winter	0	78	78	0	0	0	0
				0	13	13	0	0	0	0

*D.r. Dermacentor reticulatus*, *I.r. Ixodes ricinus*, *I.h. Ixodes hexagonus*, *F* female, *M* male, *N* nymph, *T* total

Ticks were removed from horses and cows maintained on pastures and fallow lands located near forests. Ticks from horses were collected in Mazury Lake District from two different studs. A total of 178 ticks was removed in May from one semi-wild herd (six animals) kept on a large pasture for a whole year near the University of Warsaw’s field station in Urwitałt (Fig. 1). This group was sporadically protected against ectoparasites. The second herd consisted of 32 animals used for horse riding (Dziubiele village). These horses spent nights in stables and days grazing on pasture. Tick treatment was applied on a monthly basis and grooming was carried out as a daily routine. Between June and September 2012, a total of 37 ticks were removed from these horses. In autumn 2012, 97 ticks were collected from 3 dairy cows from Dąbrowica village in the Mazowsze region. These cows were not treated against ticks and were kept on pasture during the day. The mean number of ticks per individual animal was calculated only when the number of examined animals was known.

Additionally, a total of 146 ticks collected from several European bisons shot during selective shooting in the winter of 2002/03 were included in the study (Table 1). All ticks were preserved in 96 % methanol and transferred to the laboratory at the Department of Parasitology, University of Warsaw. The species, stage and sex of adults were recorded for each tick. Ticks were then dried and weighed individually. The level of engorgement was determined on the basis of body weight.

### Level of engorgement (weight classes) of foraging ticks

Ticks found on the hosts, especially from companion animals or livestock, are usually at different stages of foraging behavior and are likely to be removed at different levels of engorgement, preventing them from the completion of their blood meal. Thus the mean weight of the ticks (females) collected from any host may not be informative enough to determine the competence of the host species for certain tick species. To minimize the negative effect of these two factors, we (1) eliminated non-engorged females from the calculation of ‘the mean weight of foraging tick’ (Table 3) and (2) established several classes of engorgement, especially to calculate the percentage of fully-engorged ready-for-reproduction females of both species.

These classes of engorgement were established based on the two tick species and two sexes, and level of engorgement:

- *Class 0: non-engorged ticks*
  For males and females, all individuals weighting below or equal to the upper 95 % CL of the mean weight of representative questing tick of each sex and species (Table 2). All ‘foraging’ *I.ricinus* males taken from the hosts also fell into this category.

**Table 2:** Abundance (mean number/100 m^2^ range) of questing ticks in two habitats during highest spring activity period (May)

Region/localization	*Dermacentor reticulatus*			*Ixodes ricinus*		
	Female	Male	Total	Female	Male	Total
*Mazowsze region*						
Warsaw						
Siekierki						
Open^b^	5.88	3.65	9.53	0	0	0
Kabacki forest						
Forest	0	0	0	0.30	0.90	1.20
Bielański forest						
Forest	0	0	0	0.90	1.30	2.20
Wołomin district						
Stoski						
Open^b^	5.5	3.0	8.5	0.13^a^	0.03^a^	0.15^a^
Kury						
Open^b^	1.94	1.06	3.00	0.64^a^	0.17^a^	0.81^a^
Dąbrowica						
Open^b^	2.92	1.61	4.53	0.17^a^	0.11^a^	0.28^a^
*Warmińsko*-*Mazurskie region*						
Mrągowo district						
Urwitałt						
Open^b^	1.63	0.96	2.60	0.04	0.16	0.20
Forest	0	0	0	0.40	0.40	0.80
Stawek						
Open^b^	0.63	0.19	0.82	0.0^a^	0.04^a^	0.08^a^
Dziubiele						
Open^b^	1.28	0.78	2.06	0.75^a^	0.15^a^	0.90^a^

^a^May 2013

^b^ *Open* fallow land
- *Class 1: slightly engorged ticks*
  For *D. reticulatus*: females weighting in the range: 0.005–0.055 g;
  For *I. ricinus*: females weighting in the range: 0.002–0.013 g.
  This class represented females which have just started their blood meal.
  The role of *D. reticulatus* males as vectors of pathogens is not clear although they have been reported being found loosely attached to the host skin (Bartosik and Buczek 2012; own unpublished observations, Dautel et al. 2006). There is no evidence that they actually take a blood meal but because we found males on the hosts weighing above the upper 95 % confidence limit of the mean weight determined for questing males, we included *D. reticulatus* males weighing above the upper 95 % confidence limit (more than 0.00480 g) in class 1.
- *Class 2: not-fully engorged females*
  For *D. reticulatus*: females weighting in the range: 0.056–0.099 g;
  For *I. ricinus*: females weighting in the range: 0.014–0.050 g.
  This class was established on the basis of Brown and Askenase (1981) [from Bartosik and Buczek (2012)] as a weight of 0.1 g is considered by these authors as a borderline weight for fully-engorged ready-for-reproduction female ticks. Females representing class 2 are probably still not engorged enough to produce and lay eggs.
- *Class 3: fully engorged females*
  For *D. reticulatus*: females weighting equal and above 0.1 g;
  For *I. ricinus*: females weighting equal and above 0.06 g;
  Engorged enough to produce and lay the eggs.

### Statistical analysis

The distribution of engorged females among 4 engorgement classes was calculated as a percentage of females in each class and was analyzed by maximum likelihood techniques based on log linear analysis of contingency tables, implemented by the software package, SPSS v. 21. Assignment to a particular class (0–3), tick species (1, 2) and host species (1–3) were fitted into a full factorial model. Beginning with the most complex model, involving all possible main effects and interactions, those combinations not contributing significantly to explaining variation in the data were eliminated stepwise (backward selection procedure), beginning with the highest-level interaction (Bajer et al. 2002). A minimum sufficient model was then obtained, for which the likelihood ratio of χ^2^ was not significant, indicating that the model was sufficient in explaining the data.

Quantitative data reflecting weight of questing and foraging ticks were expressed as arithmetic means. The weight of ticks was analyzed by multifactorial ANOVA (software package, SPSS v. 21) using models with normal errors. Tick species, sex and region of origin (Mazowsze or Warminsko-Mazurskie region) were used as the factors for analysis of the mean weight of questing ticks. Tick species, sex, host species and season were used as the factors for analysis of mean weight of foraging ticks.

Then, to estimate the borderline value for ‘not engorged’ ticks, the sum of the values of the mean weight for each sex and tick species and the value of the associated upper 95 % confidence limit was taken as the maximum borderline value for the ‘0 engorgement level class’(non-engorged ticks). Three *I. hexagonus* females and nymphs of *I. ricinus* were not taken into consideration in the statistical analyses.

## Results

### Distribution of ticks among host species

During the study period 732 ticks were collected from five host species including companion animals (dogs and cats), livestock (cows and horses) and wildlife (European bison). Three tick species were recorded: *D. reticulatus* (623 individuals; 85.1 % of all collected ticks)*, I. ricinus* 106 individuals; 14.5 %) and 3 females of *I. hexagonus* (0.4 %) from a dog (Table 1a). *Dermacentor reticulatus* was the dominant tick species found on 4 host species and constituted 86, 81, 97 and 100 % of all ticks from dogs, horses, cows and European bison, respectively. The common tick, *I. ricinus,* was the dominant tick collected from cats (94 % of all ticks) and only two *D. reticulatus* females were found on a cat (6 %).

The tick community on dogs consisted of adult *D. reticulatus* (86 %), *I. ricinus* (12.7 %) and *I. hexagonus* (1.2 %). Tick fauna from horses comprised *D. reticulatus* (81 %) and *I. ricinus* (19 %), and from cows: *D. reticulatus* (97 %) and *I. ricinus* (3 %) (Table 1a, b).

**Fig. 2:**
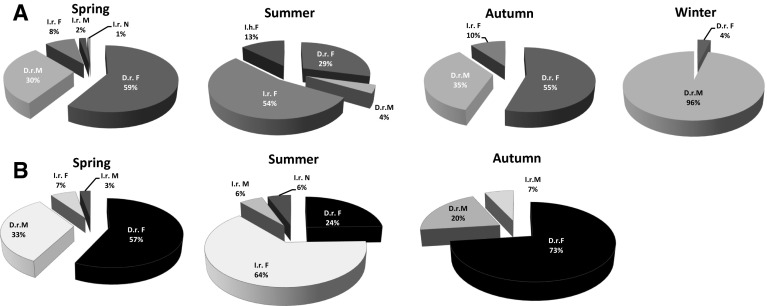
The effect of season on the tick fauna of dogs (**a**) and horses (**b**). *D.r. M* male *Dermacentor reticulatus*, *D.r. F* female *D. reticulatus*, *I.r. M* male *Ixodes ricinus*, *I.r. F* female *I. ricinus*, *I.r. N* *I. ricinus* nymphs

There were significant differences in tick community composition between seasons (Table 1a; Fig. 2a, b). The highest number of ticks was collected in the spring peak of tick activity between March and May. However, *D. reticulatus* was found on animals in every month, including during the winter season. The marsh tick was the dominant species on dogs and horses in spring and autumn (Fig. 2a, b) and was the only species recorded on hosts (dogs and bisons) during winter months. In the winter of 2002/2003, 146 ticks were collected from European bisons and all were identified as marsh ticks. In the winter of 2012/13 in Poland, winter weather lasted from late October until mid-April in both study sites, with snow cover and temperatures below 0 °C during most of this period, but there were a few spells of warmer weather (about 2 weeks each) at the end of December 2012, January and February 2013 resulting in the appearance of *D. reticulatus* on dogs in these periods (Table 1a). *D. reticulatus* males constituted the vast majority of ticks collected in winter (96 % for dogs, 92 % for European bison). In all other seasons *D. reticulatus* females were more abundant among all the ticks collected from dogs (54 %), horses (54 %) and cows (56 %) in both regions (Fig. 2a, b). The only marsh ticks removed from cats were single females in April and May. In summer months, *I. ricinus* was the main tick obtained from all examined host animals (54 % for dogs, 76 % for horses) and it was the major species collected from cats in all seasons (94 %). One *I. ricinus* nymph was removed from a dog in May, from a horse in June and from a cat in July. Additionally, 3 *I. hexagonus* females were removed from a dog in June. Cows were monitored only in the autumn season displaying high infestation with *D. reticulatus* (97 % of all collected ticks) at that time (Table 1a, b).

The mean intensity of tick infestations is presented in Table 1b. Mean intensity was calculated only if the number of sampled hosts was known. Particularly high intensity (*D. reticulatus*) was recorded on horses in May 2012, on cows in autumn 2012 and on bison in December 2002. Relatively high intensity of *I. ricinus* infestation was noted on a cat in May but it was attributable to only one infested individual. Mean intensity of tick infestation was about three ticks per dog in a group of sled dogs at the training camp in Mazury Lake District (Table 1b). However, the dogs were treated with acaricide before the camp and the majority of the collected ticks were dead.

### Comparison of questing tick abundance in two habitats

To assess the risk of infestation with two tick species, questing ticks were collected by dragging in two habitats: open (mostly abandoned fields and meadows or fallow land in city areas) and in forests, either in the Mazowsze or in Warminsko-Mazurskie regions (Table 2). The density of adult marsh ticks in open areas was relatively high, above 2 ticks/100 m^2^ in the majority of locations, with a maximum of 9.5 ticks/100 m^2^ but no marsh ticks were collected in the forests. The density of adult *I. ricinus* ticks was much lower in its typical habitat (forests), in the range of 0.8–2.2 ticks/100 m^2^, between three and seven times lower than that of *D. reticulatus* density in its typical habitat. Both tick species were found in open areas and the density of *I. ricinus* was between three and ten times lower than that of *D. reticulatus* in this habitat (Table 2).

When analyzing the proportional occurrence of marsh and common ticks on hosts (dogs and horses) in May (peak activity month for both tick species), a similar relationship was evident (Table 1a). The proportion of foraging *D. reticulatus* to *I. ricinus* was 6:1 on dogs and 7:1 on horses. However, on cats in May the proportion was reversed—1:23 in favor of *I. ricinus*.

### Mean weight of questing and foraging ticks

As we expected, tick species and sex affected the mean weight of questing adult ticks (ANOVA: tick species × sex: F_1, 164_ = 9.11, *P* = 0.003) (Table 3). The mean weight for *D. reticulatus* females was 0.00460 ± 0.00017 g and for males 0.00502 ± 0.00015 g. The mean weight of both sexes of *I. ricinus* was significantly lower than *D. reticulatus* and in this species females were significantly heavier than males (Table 3). These data supported the significant well-known differences in body size between studied species and helped to calibrate engorgement classes.

**Table 3:** Comparison of mean weight ± SEM (g) of questing and foraging adult ticks

Tick species	Sex	Questing ticks	Foraging ticks	Body mass index Foraging/questing
*Dermacentor reticulatus*	Female	0.00460 ± 0.00017	0.17457 ± 0.01133	37.95
	Male	0.00502 ± 0.00015	0.00516 ± 0.01388	1.03
*Ixodes ricinus*	Female	0.00149 ± 0.00022	0.01799 ± 0.01265	12.07
	Male	0.00076 ± 0.00021	0.00056 ± 0.02476	0.74

**Fig. 3:**
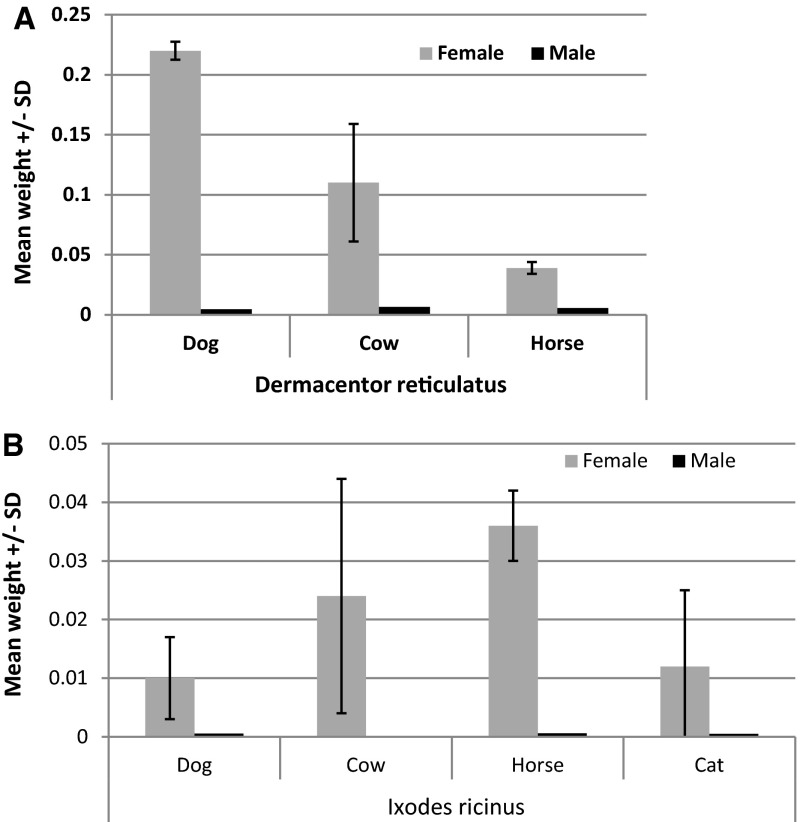
Comparison of the mean weight of female ticks collected from selected hosts: **a** for *Dermacentor reticulatus*, **b** for *Ixodes ricinus*

As all ticks found on a host must be treated as foraging despite our efforts to identify and separate a 0 class of engorgement, we calculated and analyzed the mean weight of *D. reticulatus* and *I. ricinus* adult ticks collected from certain host species. Arithmetic means are presented in Table 3. Questing and foraging males of both tick species had similar weights (index of engorgement: 0.7–1.03; Table 3), without any significant differences between the host of origin (Fig. 3a, b). Females of both tick species demonstrated a significant increase in body mass when foraging on hosts and overall engorgement success was higher in the case of *D. reticulatus* females in comparison to those of *I. ricinus* (index of engorgement: 37.95 and 12.07 for *D. reticulatus* and *I. ricinus*, respectively; Table 3).

Ticks collected from European bison in the winter of 2002/2003 were excluded from the statistical analysis to avoid a ‘time effect’ on body mass. Multifactorial ANOVA revealed a significant 3-way interaction of host species, tick species and sex on mean weight of foraging ticks (F_1, 579_ = 3.92, *P* = 0.048). This interaction is illustrated in Fig. 3a, b for the major host species. For *D. reticulatus* females the highest mean weight was recorded for ticks foraging on dogs and cows, and was relatively lower for ticks feeding on horses. For *I. ricinus* females, the highest mean weight was recorded for ticks foraging on horses and cows.

### Comparison of female engorgement on different hosts

**Fig. 4:**
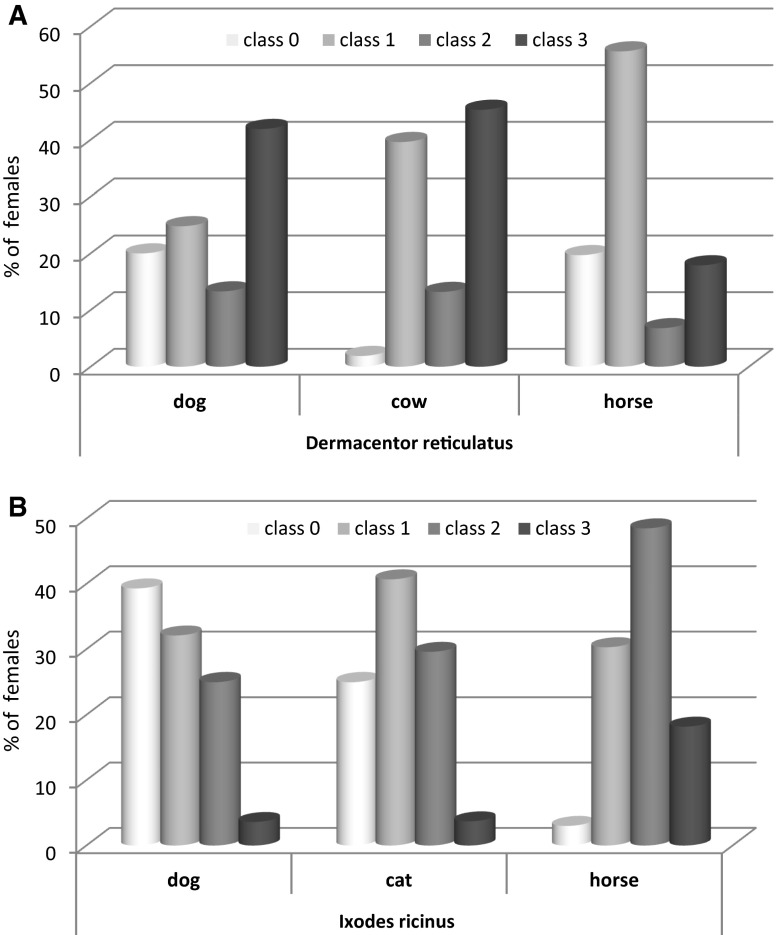
Distribution of female ticks collected from selected hosts, among engorgement classes as defined in the Materials and Methods section: **a** For *Dermacentor reticulatus,* **b** For *Ixodes ricinus*

Finally, to assess host competency, we analyzed the distribution of different engorgement classes of tick females on hosts. The minimal sufficient model comprised one significant interaction of host species, tick species and engorgement class ($\chi^{ 2}_{ 1 2}$ = 31.72, *P* = 0.002). This interaction is presented in Fig. 4a, b. Two *D. reticulatus* females collected from cats were engorged (class 3) and the vast majority of ticks collected from bison were males. For these reasons we have not included the data from cats and bisons in the analysis and figure. In accordance with analysis of mean weight, for *D. reticulatus* females, the highest rate of fully engorged females (class 3) was noted for dogs and cows, and it was lower for horses. In contrast, for *I. ricinus* females, the highest rate of fully engorged females (class 3) was noted for horses (Fig. 4b).

Among 334 *D. reticulatus* males collected from five host species, 149 (44.6 %) fell in class 1 of engorgement. Males in this class constituted 19, 57, 58 and 78 % of males collected from European bisons, horses, dogs and cows, respectively.

## Discussion

The main aim of our study was to determine the composition of the tick community on domestic companion and livestock animals in two endemic areas for *I. ricinus* and *D. reticulatus* and to identify competent hosts for the marsh tick. The dominant species found on cows, horses and dogs was *D. reticulatus,* except for the summer, when *I. ricinus* was mainly collected from all host species. The highest number of ticks was obtained in spring, and subsequently in the autumn. In spring, the abundance of questing *D. reticulatus* ticks was much higher than *I. ricinus* in their typical habitats, constituting a higher risk of infestation.

The tick fauna on livestock and dogs depends mainly on the geographical location, as different tick species inhabit different continents, and the geographical range for different tick species determines their occurrence on hosts. In Poland, for example, *R. sanquines* is an accidental, imported species, but this tick species is dominant on dogs in southern Europe around the Mediterranean basin. Thus, comparison of the composition of the tick community even among cattle or dogs is difficult and we only discuss here data for the three detected tick species in European dogs, excluding Mediterranean countries. In our study, *D. reticulatus* comprised 86 % of all ticks collected from dogs and this is definitely the highest proportion of this species found to date on dogs in Europe. In an earlier study based in Warsaw, this species dominated over *I. ricinus* among ticks from dogs (65 vs. 35 %; Zygner and Wedrychowicz 2006). In the Ukraine, near Kiev, among 52 ticks, *D. reticulatus* constituted 63 % (Hamel et al. 2013). Both eastern and central Poland and Ukraine are inhabited by the eastern population of this tick (Karbowiak 2009, 2014) and apparently the risk of infestation with this species is high in this area. In other endemic regions for *D. reticulatus* in Central Europe, this tick species shows comparable frequency to *I. ricinus* on dogs (i.e. 45 % of ticks in Germany, Beck et al. 2014; 49% of ticks in Hungary, Földvári and Farkas 2005). In a recent study in eastern Austria, *D. reticulatus* constituted 15 % of all ticks, but was dominant in early spring and late autumn (Duscher et al. 2013). In all countries, in which *D. reticulatus* is a significant component of the tick fauna on dogs, an increased risk of canine babesiosis is expected, as this tick species is the main vector of *B. canis* (Rar et al. 2005; Zygner et al. 2008, Schaarschmidt et al. 2013; Mierzejewska et al. 2012). The marsh tick is still rare in the UK and in Belgium (0.6–0.8 %) but has been established recently as a permanent feature of the local tick fauna on dogs in these countries (Smith et al. 2011, Claerebout et al. 2013). Interestingly, in southern Poland (Rymanów, Podkarpackie region), *D. reticulatus* was not found among 236 ticks from dogs, where only *I.ricinus* and *I. hexagonus* were identified (Kilar 2011), but this only confirms the existence of the gap between the eastern and western populations of this tick species (Fig. 1, Karbowiak 2009).

In our study, the common tick *I. ricinus* constituted only 13 % of ticks collected from dogs and this is the lowest percentage of this species found on dogs in Europe. The percentage of *I. ricinus* was 36, 43 and 46 %, in recent studies in Ukraine, Hungary and Germany, respectively (Hamel et al. 2013; Földvári and Farkas 2005; Beck et al. 2014). The highest percentage of this tick species was found among dogs from the UK (52–72 %; Smith et al. 2011; Ogden et al. 2000), Belgium (76 %; Claerebout et al. 2013), Austria (76 %; Duscher et al. 2013) and also in southern Poland (89 %, Kilar 2011). Because of the much higher abundance of *D. reticulatus* compared with *I. ricinus* in open habitats (which are more often used by dogs and livestock) where both species are sympatric, and generally similar densities noted for *I. ricinus* right across Europe (Welc-Faleciak et al. 2014), with further expansion of the marsh tick, the overall risk of tick infestation and consequently, of exposure to canine TBDs is likely to increase up to 6–7 times. Already, some evidence for a rapid recent increase in the risk of contracting canine babesiosis, as a result of the expansion in the range of *D. reticulatus,* has been noted in central Poland (Bajer et al. 2014b). Interestingly, social perception of ticks and TBDs in dogs (and the proper use of repellents/acaricides) is much higher in central and eastern Poland than in southern and western regions, most likely due to the high abundance of *D. reticulatus* (Bajer et al. 2014a, b).

*Ixodes hexagonus* constituted a significant component of the tick fauna on dogs in the UK (22–39 %; Smith et al. 2011; Ogden et al. 2000) and in southern Poland (10.6 %; Kilar 2011) but was rare among dogs from our study and studies from Hungary and Austria (0.1–0.4; Duscher et al. 2013; Földvári and Farkas 2005) and was not recorded in the Ukraine (Hamel et al. 2013).

Analyses of the proportional distribution of common and marsh ticks collected from livestock and dogs, and from their habitats (1:6, 1:7), revealed no marked host selection for *D. reticulatus* and supported host competency of cattle, horses and dogs for this host species. Higher densities of questing *D. reticulatus* than *I. ricinus* (adults) may be related to the much shorter life cycle of *D. reticulatus* compared with *I. ricinus* (1-year vs. 2-year long life cycle)(Zahler and Gothe 1995; Cochez et al. 2012). Interestingly, although *I. ricinus* clearly dominated on cats and these host species are believed to be too small to feed *D. reticulatus*, we found two fully engorged females on cats, so cats can be considered as hosts for *D. reticulatus*, though less preferred. Host competence of livestock and dogs for *D. reticulatus* was supported by the finding of a significant rate of fully-engorged ready-for-reproduction females on these hosts and a significant increase in mean body mass of ticks feeding on them. However comparison of female engorgement classes revealed the highest rate of fully engorged females only on cattle (45 %) and dogs (42 %). The much lower proportion of fully engorged females collected from horses (18 %) may be attributable to the higher number of well-cared-for horses involved in the study. Comparison of mean tick numbers showed a lower intensity of infestation on horses which were groomed and received regular acaricide treatment (Dziubiele, June, October, 84 % of all horses) compared with those which were not treated/groomed (Urwitałt, May).

Dogs were the most numerous group of animals included in this study and were characterized by the most uniform distribution of *D. reticulatus* females among engorgement classes, but nevertheless the proportion of females in class 3 was the highest. A case of an unprotected bitch from Tłuszcz presenting in April 2014 at the veterinary clinic with a massive tick infestation showed the full potential of those animals as competent hosts for marsh ticks. The dog lived in an endemic region and did not get anti-tick treatment in time due to the atypically early and warm spring. Veterinarians removed altogether 76 ticks from this dog, including 66 *D. reticulatus* (39 females and 27 males) and 10 *I. ricinus* (7 females and 3 males). At this time 35 females (90 %) were in the highest class of engorgement (mean body weight 0.398 g) while only 4 (10 %) in the second class of engorgement (mean body weight 0.0801 g). No females from any lower classes were detached from this dog.

In our study *D. reticulatus* ticks were found on dogs through the whole year, including the winter season and were also collected during the winter from European bison. The occurrence of *D. reticulatus* on hosts and on vegetation in winter has been previously recorded by other authors, contrary to *I. ricinus*, which is generally absent in winter months. Questing marsh ticks have been collected in winter in Germany (Dautel et al. 2008) and Poland (Bartosik et al. 2011; Buczek et al. 2014). The presence of marsh ticks on the European bison from Białowieża primeval forest in the winter of 1992–2000 was reported by Izdebska et al. (2001) and Karbowiak et al. (2003). Other host species that have been recorded as infested in winter include moose, red deer and wild boar (Izdebska et al. 2001). Both sexes of ticks were collected, but male ticks represented the majority, as in our study. Several tick females were fully engorged and laid eggs in the laboratory, establishing that *D. reticulatus* is active and most likely able to transmit pathogens also in winter time (Karbowiak 2009). Based on the findings of other authors and our results we have confirmed that year-round activity is the normal behavior of *D. reticulatus* and consequently it is likely to play an important role in the circulation and maintenance of tick-borne pathogens throughout the year. Therefore, whole year repellent/acaricide protection for dogs should be provided in endemic regions, especially in late autumn, during mild winters and in early spring, as *D. reticulatus* activity in these periods may be responsible for *B. canis* infections.

The low numbers of ticks collected from cats is probably linked to their behavior (effective self-grooming; Marchiondo et al. 2013) and the specific type of animals brought to the veterinary clinic. It is more likely that cats with a lower risk of ectoparasite infestation (kept indoors or those that are let outside only for a short period at a time) were brought to the veterinary clinic. The majority of ticks collected from cats were *I. ricinus,* only two female *D. reticulatus* being recorded. Higher infestation with *I. ricinus* may be explained either by host selection (adult *D. reticulatus* are believed to feed on large mammals) or the type of acaricide employed by owners. Most of the spot-on drugs used on cats in Poland are based on fipronil. This agent seems to be less effective against the common tick (Leschnik et al. 2013; Beck et al. 2014), but is the most effective acaricide for the marsh tick and should be treated as the reference acaricide in *D. reticulatus* endemic areas (Bajer et al. 2014b, Beck et al. 2014).

Males of *D. reticulatus* constituted over 90 % of all ticks collected during the winter. A similar sex bias in this season has been observed in other studies (Karbowiak 2009; Izdebska et al. 2001; Bartosik et al. 2011; Buczek et al. 2014), but the role of males that may remain on hosts for several weeks, remains poorly understood. Bartosik and Buczek (2012) suggested that the presence of male *D. reticulatus* was necessary for females to cease feeding and to enable their fertilization by engorged males. In our study the bodies of detached *D. reticulatus* males were slightly enlarged, but the mass index did not show significant differences between foraging and questing males (1.03) and the mean weight of *D. reticulatus* males collected from different hosts was similar, suggesting that it is unlikely that males take a proper blood meal on any of the hosts in the study.

Comparison of the mean body weight of foraging female common and marsh ticks showed that for *D. reticulatus* the mass index was more than 3 times higher than *I. ricinus* (37 vs. 12 times). A higher volume of ingested blood may increase the success of pathogen transmission between host and vector, thus *D. reticulatus* seems to be a greater threat than the common tick for livestock and domestic animals lacking proper acaricide/repellent treatment.

## Conclusions

In eastern and central regions of Poland endemic for marsh ticks, this tick species constitutes the main risk of tick infestation in livestock and dogs throughout the year. Domestic and farm animals are competent hosts for *D. reticulatus*, enabling the completion of the tick life cycle and promoting its expansion.
